# The epidemiology of all-cause and rotavirus acute gastroenteritis and the characteristics of rotavirus circulating strains before and after rotavirus vaccine introduction in Yemen: analysis of hospital-based surveillance data

**DOI:** 10.1186/s12879-015-1165-8

**Published:** 2015-10-13

**Authors:** Salem M. Banajeh, Basheer A. Abu-Asba

**Affiliations:** Faculty of Medicine & Health Sciences, Sana’a University, Sana’a, Yemen; Surveillance Unit, Ministry of Public Health, Sana’a, Yemen

**Keywords:** Rotavirus vaccine, Severe rotavirus hospitalization, Severe dehydrating diarrhea, Rotavirus strains, Yemen

## Abstract

**Background:**

Rotavirus (RV) vaccine was added to Yemen’s childhood vaccination schedule in late 2012. We evaluated the effect of vaccination on the epidemiology of acute gastroenteritis (AGE) and the characteristics of circulating RV strains.

**Methods:**

Surveillance data was obtained at two sentinel hospitals from 5,691 children with acute gastroenteritis (AGE) who were under 5 years of age. Data collected before (2007–2011) and after (2013–2014) RV vaccine introduction were retrospectively analyzed. Treatment outcome, presence of severe dehydration, and the proportion of all-cause AGE due to RV-antigen positive AGE were calculated for each period and compared. Binominal generalized linear models were used to calculate adjusted odds ratios (ORs) and 95 % confidence intervals (CIs). We also compared severe RVAGE and severe dehydration proportions in hospitalized children with severe AGE and characterized RV circulating strains in available specimens from the two periods.

**Results:**

Before RV vaccination, mean RVAGE prevalence peaked in October (58.8 %), November (69.5 %), and December (56.4 %). In 2013–2014, the variation became less defined, with only a few RVAGE cases. The average annual prevalence of severe RVAGE needing hospitalization was 42.9 % in 2007–2011, decreased to 21.1 % in 2013, and to 18.5 % in 2014, representing declines of 50.8 % (95 % CI: 36.4–65.0) and 56.9 % (95 % CI: 42.1–70.5). The proportion of children <12 months of age with all-cause AGE decreased significantly after introduction of RV vaccination (58.7 % vs. 62.3 %; *p* = 0.042), severe dehydration decreased by 50 % (14.7 % vs. 21.7 %; OR = 0.501, *p* < 0.0001), and RVAGE proportion decreased by 48 % (19.9 % vs. 41.6 %; OR = 0.52, *p* < 0.0001). The proportion of severe RVAGE in hospitalized patients decreased by 67 % (20.1 % vs. 43.5 %; OR = 0.33, *p* < 0.0001), and severe dehydration decreased by 58 % (17.2 % vs. 33.1 %; OR = 0.42, *p* < 0.0001). Non-RV AGE prevalence significantly increased, with ORs of 2.8–3.1 in favor of non-RV AGE in 2013–2014. Analysis of 128 available stool specimens revealed that circulation of the G1 genotype did not change following vaccination (33.3 % vs. 41.3 %; *p* = 0.366). G2 significantly decreased in 2013–2014 (4.2 % vs. 42.5 % *p* = 0.0001), and G9 increased (29.2 % vs. 6.3 %; *p* = 0.001). G1P[8] and G2P[4] remained prevalent, and G9P[8] and G9P[4], which were not detected in the pre-vaccine period appeared in 2013–2014. G and [P] mixed genotypes became more prevalent in 2013–2014. It is not known if this predominance is related to the vaccine introduction or attributable to normal genotype fluctuations.

**Conclusions:**

Rotarix substantially reduced the prevalence of RVAGE, with a 67 % reduction of severe RVAGE hospitalizations, and over 50 % reduction of diarrhea with severe dehydration. Circulation of RV G and [P] mix strains was significantly increased in 2013–2014 and needs continuous monitoring.

## Background

In developing countries, diarrhea remains one of the most frequent causes of death among children under 5 years of age. Rotavirus (RV) is the most common cause of severe diarrhea in infants and young children and the major cause of diarrhea-related child mortality. It accounts for more than 2 million hospital admissions annually [1–3]. The World Health Organization (WHO) recommends the introduction of rotavirus vaccine in national immunization programs especially in developing countries with high childhood mortality [4, 5]. Yemen is one of the least developed countries and is the only low-income country in the Arabian Peninsula. It has a population of 25.2 million, 16.1 % of whom are under 5 years of age. Health information published in 2014 by the WHO estimates that 47 % of children in Yemen under 5 years of age are stunted and 16 % are wasted [6]. The data also indicates that only 27.8 % of children who developed diarrhea received oral rehydration therapy and estimates infant and under 5-year mortality rates are 40.4 and 51.3 per 1000 live births, respectively [6].

Acute gastroenteritis (AGE)-related morbidity and mortality is a major health problem in Yemen. A recent report on the global, regional, and national causes of child mortality estimated that in 2013, 16.8 % of 19 607 deaths from all causes in children 1–59 months of age was diarrhea related. The corresponding estimate for 2007 was 21.2 % of 26,800 deaths, indicating a reduction of only 4.4 % over a 7-year period [7]. Data on the burden of rotavirus acute gastroenteritis (RAVGE) in Yemen are scarce. Only one hospital-based study was published before the introduction of rotavirus vaccine, In that study, Kirby et al. reported that 27 % of children from 1 to 5 years of age who were treated for AGE in the emergency and outpatient departments of two hospitals in Sana’a had rotavirus, and 10 % had norovirus infections [8]. However, the study was limited by its geographical restriction to one city located in the northern highlands region of Yemen.

There were three study objectives. The first was to examine trends in the annual prevalence of RVAGE in children hospitalized with severe AGE. The second was to compare the proportions of RVAGE, WHO-defined severe dehydration, and the treatment outcomes of children younger than 5 years of age who were treated for all-cause AGE at the sentinel hospitals or were hospitalized with severe dehydrating diarrhea. The third was to assess and examine the prevalence, characteristics and diversity of the circulating rotavirus strains, before and after introduction of RV vaccine in Yemen’s national immunization program.

## Methods

In 2004, the Ministry of Public Health in Yemen implemented surveillance of AGE (including RVGE) in children less than 5 years of age at sentinel hospitals. The surveillance unit is supported technically and financially by the WHO Eastern Mediterranean Regional Office (EMRO), which includes surveillance training for hospital staff. The standard operating procedures (SOPs) used by the Rotavirus gastroenteritis surveillance network in the EM Region are described in the WHO generic protocol for rotavirus surveillance [9].

### Study sites

The study evaluated surveillance data collected from two sentinel hospitals. The first was the Al --Sewedy hospital located in Taiz city, serving the population of Taiz and the surrounding rural areas. The Taiz governorate has a population of 4 million and is located in the mountainous midland region of Yemen at an altitude of 1400 m above sea level. The second center was Al-Wehdah hospital located in the southern city of Aden, which serves a population of 1,400,000, including Aden and the adjacent southern governorates. Aden is located along the coast of the Arabian Sea. Each hospital has a capacity of more than 600 beds, walk-in outpatient units receiving patients on work days, and 24-h emergency rooms. Surveillance began at Al-Sewedy Hospital in 2007, and at Al-Wehdah Hospital in 2008. Following the WHO–EMRO guidelines, a trained surveillance officer at each hospital collects information on children who visit the hospital because of AGE and submits standard case report forms to the surveillance unit every month. The reports are checked for accuracy and missing data are obtained by communication with the surveillance officer at the sentinel hospital. Data are recorded in Microsoft Office Excel 2007 software.

Rotarix™ vaccine (GlaxoSmithKline Biologicals, Belgium) was introduced in the national immunization program of Yemen in October 2012 with support from the Global Alliance for Vaccines Initiative (GAVI). Rotarix, is a monovalent live attenuated human rotavirus vaccine based on the G1P[8] strain. It is given orally as two doses at 6 and 10 weeks of age, and is free of charge to all children in Yemen. The national vaccination coverage rates for the second dose of Rotarix was 23 % in 2012 and 90 % in 2013. The coverage rates in Taiz and Aden were 25 and 30 % in 2012 and 76 and 87 % in 2013, respectively [10].

### Ethical approval

The ethical review committee of the Yemen Ministry of Public Health advised that ethical approval for this study protocol was not needed as it was considered to be a part of an ongoing national surveillance activity. All study procedures constituted standard case management. This was a retrospective analysis of data collected from the sentinel hospitals, and patients were enrolled only after signed informed consent had been obtained from their parents or legal guardians. The data did not include names of individual patients, and the authors did not follow or contact the parents following enrolment.

### Study Population

Children less than 5 years who attended the sentinel hospital outpatient unit, emergency room, and/or were admitted as inpatients with a primary diagnosis of AGE were included in the study after informed consent was obtained.

### Definitions

AGE (in children less than 5 years of age) was defined as at least three watery or looser than normal stools in a 24-h period and of less than 7 days duration, and/or two or more vomiting episodes that were unexplained by other reasons. Dehydration status was scored as follows. Severe dehydration (1) required two or more of the following signs: lethargy or unconsciousness, sunken eyes, inability to drink, and skin pinch recovers very slowly. Some dehydration (2) required two or more of the following signs: restlessness or irritability, sunken eyes, thirst or drinking eagerly, and skin pinch goes recovers slowly. No dehydration (3) was noted in children with none of the above signs).

### Study design

This was a retrospective observational study based on AGE surveillance data collected prospectively during the pre-RV vaccine period from January 2007 to December 2011, and in the post-RV vaccine period from January 2013 to December 2014. Surveillance data collected during 2012, the year in which the RV vaccine was introduced, were excluded from the analysis.

### Data collection

Information on patient age (months), sex, and place of treatment (outpatient clinic, emergency room, or inpatient facility), plus clinical information including duration of symptoms, diarrhea and/or vomiting (days), the number of episodes per day before the visit, and whether fever was present, was collected. Details of dehydration status (severe, some, or none), treatment given, duration of hospitalization, and treatment outcome on discharge were also collected. Stool specimens were obtained from children enrolled in study not later than 7 days after the onset of the illness and within 48 h of enrollment. Specimens were placed in labeled screw-top containers and stored at 4 °C. Rotavirus antigen was detected in the specimens by enzyme immunoassay (EIA) carried out in the hospital laboratory using WHO-approved kits (IDEIA™, DAKO Diagnostics, United Kingdom). Rotavirus-positive specimens were stored at −20 °C, according to SOP guidelines, until sent to the Regional Rotavirus Reference Laboratory in Cairo, Egypt for G and P genotyping.

### Date management and statistical analysis

The surveillance data from all children included in the study were imported from Microsoft Office Excel 2007 into SPSS software, version 20 (IBM, Armonk, NY, USA). We used descriptive statistics to calculate the annual prevalence of severe RVGE in children hospitalized with all-cause severe AGE from 2007 to 2014 and the percentage decline in prevalence in 2013 and 2014 compared with the average annual prevalence in the pre-vaccine years. We also examined changes in the distribution of emergency unit visits and hospitalizations for all-cause AGE and RVGE before and after vaccine introduction by comparing the percentage reductions and 95 % confidence intervals of the annual median occurrence of these events. Pre- and post-vaccine patient characteristics were compared using Pearson’s chi-square test for categorical variables and student’s-*t* test for continuous variables with equal variance not assumed.

### Measurement of the core study outcomes

We measured the effect of RV vaccine introduction on all-cause AGE by comparing the proportions of patients with severe dehydration, the prevalence of RVAGE, and treatment outcomes (core study outcomes) obtained during the two study periods. We used binominal generalized linear models with a probit link function. The pre- and post-vaccine periods were dependent categorical variables with the post-vaccine period considered as a response and the pre-vaccine period as the reference. The core study outcomes were introduced separately in the model. Each study outcome was adjusted for the following predictors: age, gender, duration of symptoms (days), number of diarrhea episodes per day, and hospital site. The statistical procedure computed the main effect of these cofounders expressed as an adjusted exponential (Beta) with its 95 % Wald confidence interval (95 % Wald CI) as well as the *p* value. We also compared the proportions of patients with severe RVAGE and severe dehydration among those hospitalized for all-cause severe AGE, and the proportions of different RV circulating strains in the pre- and post-vaccine periods. The statistical significance level was set at *p* < 0.05.

## Results

### Surveillance data of all participants

During the study period from January 2007 to December 2014, 5691 children with all-cause AGE and under 5 years of age visited the sentinel hospitals and were included in the analysis. Most children were young. The mean age was 12.2 months, and 3425 (60.2 %), were under 12 months of age. The mean duration of symptoms, vomiting, diarrhea, and diarrhea episodes/day were 3.1, 2.9, 3.2, and 6.4, days respectively. A total of 2886 children (50.7 %) were hospitalized with severe dehydrating AGE, 2308 (40.6 %) visited the emergency room, and 479 (8.7 %) visited the outpatient unit. Stool specimens were collected from 5495 children (96.6 %). A total of 1940 specimens (35.3 %) were RV-antigen positive by EIA and 1210 of those (62.4 %) were obtained from children less than 12 months of age. Stool specimens from 2747 (95.2 %) of the 2886 children who were hospitalized with severe AGE (*n* = 2886), were tested, and of those, 1005 (36.6 %) were RV positive. The annual prevalence of hospitalized severe RVAGE remained stable with an average of 42.9 % from 2007 to 2011. The prevalence dropped to 21.1 % in 2013 and to 18.5 % in 2014 (Table 1); representing percentage decreases of 50.8 % (95 % CI: 36.4–65) and 56.9 % (95 % CI: 42.1–70.5), respectively.

**Table 1:** Acute Gastroenteritis: hospitalization, NO(%) of cases that have stools tested for RV antigen and annual prevalence of severe RVAGE from 2007 to 2014

Year of study	AGE hospitalization	Cases stool tested	Cases positive RV + ve	% Annual prevalence
	N	N (%)		(RVGE)
2007	480	480 (100)	210	43.8
2008	239	239 (100)	104	43.5
2009	441	382 (86.6)	198	51.8
2010	514	465 (90.5)	194	41.7
2011	288	269 (93.4)	90	33.5
2012	236	236 (100)	73	30.9
2013	421	417 (99)	88	21.1
2014	264	259 (98)	48	18.5

### Pre- and post-vaccination periods

#### Seasonal variation

**Fig. 1:**
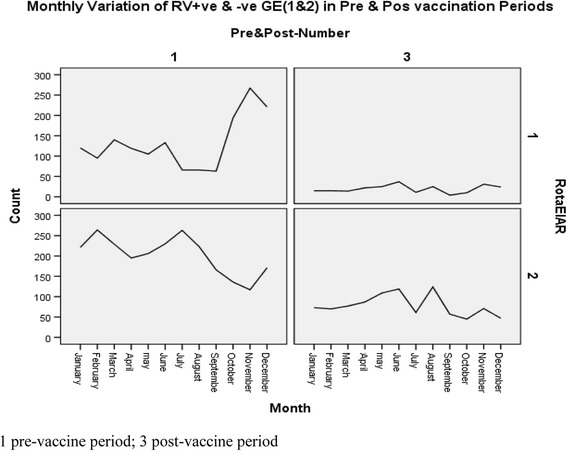
Monthly variation of RV + ve & RV –ve AGE: Pre and Post vaccination periods

In the pre-vaccine period, the proportion of children with RVAGE followed a well-defined monthly pattern that peaked in October (58.8 %), November (69.5 %), and December (56.4 %) and was lowest in July (20 %), August (22.8 %), and September (27.5 %). In the post-vaccine period, the seasonal variation became blunted and less well defined with only a few cases of RVAGE detected (Fig. 1).

#### All-cause AGE

Compared with the pre-vaccine period, the proportion of children under 12 months of age with all-cause AGE was significantly smaller in the post-vaccine period (62.3 % vs. 58.7 %, *p* = 0.042), but a significantly larger proportion presented with vomiting (88 % vs. 93.1 %; *p* < 0.0001) and a significantly smaller proportion presented with fever (63.6 % vs. 45.1 %; *p* = <0.0001) (Table 2). The mean ± SD duration (days) of symptoms and of diarrhea before seeking treatment was both longer in the post-vaccine period (2.98 ± 1.67 vs. 3.33 ± 1.58; *p* < 0.0001, and 3.02 ± 2.12 vs. 3.40 ± 1.89; *p* = 0.0001, respectively). There was no significant difference in the duration of hospital treatment (Table 2). Severe dehydration decreased significantly from 21.7 % of children with all-cause AGE (848/3902) in the pre-vaccine period to 14.7 % (168/1144) in the post-vaccine period (unadjusted OR 0.64, 95 % CI: 0.54–0.77; *p* = 0.0001). The median annual number of visits for all-cause AGE decreased by 26 % (95 % CI: 23–29), from 800 in the pre-vaccine to 591 in the post-vaccine period. Emergency room visits decreased from 358 to 180, and hospitalizations decreased from 444 to 342, representing median reductions of 49.7 % (95 % CI: 44.6–54.9), and 23 % (95 % CI: 19.3–27.1), respectively.

**Table 2:** Baseline characteristics: all-cause acute gastroenteritis: pre and post rotavirus vaccine introduction

Variable No(%)	Pre-vaccine period	Post-vaccine period	OR (95 % CI)	*P* value
	*N* = 3973	*n* = 1182	(post-vaccine cohort)	
Age group				
< 12 months	2476(62.3)	694(58.7)	0.87(0.76–0.99)	0.042
12–23 month	1042(26.2)	310 (26.2)	0.99(0.86–1.15	0.887
> 23 months	455(11.5	178 (15.1	1.35(1.12–1.63)	0.001
Females	1557 (39.2)	449 (38.0)	0.96(0.87–1.07)	0.456
Males	2416 (60.8)	733 (62.0)		
Vomiting	3495(88.0)	1101 (93.1)	1.65(1.34–2.04)	<0.0001
Febrile	2528(63.6)	533(45.1)	0.56(0.51–0.62)	<0.0001
Age (months)				
Mean(SD)	11.74(9.44)	12.93(11.20)		0.001
Duration of symptoms days				
Mean(SD)	2.98(1.67)	3.33(1.58)		<0.0001
Diarrhea episodes/day				
Mean(SD)	6.77(4.34)	5.57(3.41)		<0.0001
Diarrhea duration(days):				
Mean (SD)	3.02(2.12)	3.40(1.89)		<0.0001
Hospital treatment (days)				
Mean (SD)	2.23(1.67)	2.09(2.44)		0.690
Severe dehydration	848/3902 (21.7)	168/1144 (14.7)	0.641(0.535–0.767)	<0.0001
Rotavirus gastroenteritis	1528/3676 (41.6)	233/1173 (19.9)	0.45(0.38–0.51)	<0.0001
Treatment outcome	*N* = 3899	*N* = 1161		
Successful treatment	3547(91.0)	957(82.4)		0.0001
Deaths	27(0.7)	13(1.1)		0.177
Referred for intensive care	60 (1.5)	8(0.7)		0.037
Not known	265(6.8)	183(15.8)		0.0001

### Severe all-cause AGE

In the pre-vaccine period, 1968 children required hospitalization for severe all-cause AGE but only 683 were hospitalized in the post-vaccine period. The percentage of children less than 12 months of age who required hospitalization did not significantly decrease, however [1208 (61.4 %) vs. 414 (60.6 %); *p* = 0.939]. Severe dehydration decreased significantly from 648/1960 children (33.1 %) in the pre- to 117/681 children (17.2 %) in the post-vaccine period, a 58 % reduction (OR = 0.42, 95 % CI: 0.34–0.52; *p* < 0.0001). The number of children with severe RVAGE decreased by 67 % after introduction of Rotarix vaccine from 796/1828 children (43.5 %) to 136/675 children (20.1 %), an unadjusted OR of 0.33 (95 % CI: 0.27–0.40; *p* < 0.0001).

### Measurement of the core study outcomes

Treatment outcome proportions, severe dehydrating diarrhea, and the prevalence of RVAGE within all-cause AGE in the pre- and post-vaccination periods were compared using a generalized linear binominal model with a probit link function controlling for all possible predictors. The analysis showed that successful treatment outcome was not significantly different in the two periods. Similarly, AGE-related deaths before and after the introduction of rotavirus vaccine did not differ significantly. However, referral for intensive care was significantly less frequent in the post-vaccine period (Tables 2 and 3). Severe dehydrating diarrhea, and the overall prevalence of RV antigen-positive, showed statistically significant reductions approaching 50 and 48 % in 2013–2014, respectively (Table 3).

**Table 3:** Core study outcomes during pre & post-RV vaccination periods: binominal generalized linear models with probit link function

Pre & Post vaccination	Unadjusted Exponential (B)		Adjusted Exponential(B)^b^	
Periods N(%)	(95 % Wald CI)^a^	P	(95 % Wald CI)^a^	P
Treatment outcome				
Successfully treated				
Pre: 3547/3899 (90.9)	1		1	
Post: 957/1161 (82.4)	0.640 (0.569–0.720)	0.0001	0.995 (0.808–1.227)	0.964
Referred for intensive care				
Pre: 60/3899 (1.5)	1		1	
Post: 8/1161 (0.7)	0.442 (0.295–0.660)	0.0001	0.615 (0.400–0.946)	0.027
Deaths				
Pre 27/3899(0.7)	1		1	
Post 13/1161 (1.12)	0.801 (0.526 -1.219)	0.299	0.772 (0.478–1.248)	0.291
Severe dehydrating diarrhea				
Pre: 848/3902 (21.7)	1		1	
Post: 168/1144 (14.7)	0.64 (0.54–0.77)	0.0001	0.501 (0.416–0.602)	0.0001
Rotavirus gastroenteritis				
Pre: 1528/3676 (41.6)	1		1	
Post: 233/1173 (19.9)	0.45 (0.38–0.51)	0.0001	0.523 (0.476–0.574)	0.0001

^a^Confidence Interval

^b^Adjusted for age group, gender, hospital site, symptoms duration(days), and diarrhea episodes duration(days)

### RVAGE

Our study showed that there was a significant 48 % decrease in the prevalence of RVAGE in the post-vaccine period (Table 3). In the post-vaccine period, the proportion of children under 12 months of age with RVAGE (57.1 %, 133/233) was also significantly reduced when compared with the pre-vaccine period (65.6 %, 1002/1528) giving an OR of 0.718 (95 % CI: 0.543–0.949; *P* = 0.004). The median annual number of RVAGE hospitalizations was 194 in the pre-vaccine period compared with 68 in 2013–2014, a 65 % reduction (95 % CI: 58–71). The number of emergency unit visits decreased by 58 % in the post-vaccine period to 28.8 % (67/233) compared with 44 % (672/1528) during the pre-vaccine period, an OR of 0.42 (95 % CI: 0.323–0.533); *p* = 0.0001). Among those hospitalized with severe RVAGE, severe dehydration was also significantly reduced from 34.7 % (276/795) before vaccination to 19.3 % (26/135) in 2013–2014, an OR of 0.45 (95 % CI: 0.29–0.71; *p* < 0.0001). In contrast, the proportions of all non-RV AGE patients, patients who were hospitalized, and/or attended the emergency room were all significantly greater in 2013–2014 compared with the pre-vaccine period. For all non-RV AGE patients, the proportions were 80.1 % (940/1173) and 59.4 % (2239/3767), an OR of 2.8 (95 % CI: 2.4–3.2). For hospitalized patients, they were 79.9 % (539/675) and 56.5 % (1032/1828), an OR of 3.1 (95 % CI: (2.5–3.8), and for emergency unit visits they were 81.3 % (292/359) and 60.8 % (1041/1713), an OR of 2.8 (95 % CI: 2.1–3.7).

### Prevalence and genotype distributions of circulating rotavirus strains

Genotyping was available for 80 pre-vaccination and 48 post-vaccination RV strains (Table 4). The majority—74/80 of the pre-vaccination strains (83 %) and 40/48 post-vaccination strains (92.5 %)—were isolated from children less than 24 months of age. The most prevalent G types in the pre-vaccination period were G1 (33/80, 41.3 %) and G2 (34/48, 42.5 %). The percentage of G1 did not change in the post-vaccine period (16/48, 33.3 %) but G2 was dramatically reduced (2/48, 4.2 %). G9 increased from 5/80 strains (6.3 %) before, to 14/48 strains (29.2 %) after, RV vaccine introduction. P genotypes showed similar variation, with P[4] accounting for 33.8 % of the pre-vaccination strains, but became less frequent, comprising 14.3 % of the post-vaccine strains. P[8] strains increased in frequency from 26.3 to 51 % after Rotarix introduction. We observed increasing frequencies of mixed G and P genotypes circulating in the post-vaccine period that had previously been extremely uncommon. These included G1G3, G2G9, and G3G9, accounting for 20.8 % (10/48) and P[4]P[8], P[6]P[8], and P[8]P[10], accounting for 22.9 % (11/48) of the post-vaccine strains. These genotypes accounted for only 6.3 % (5/80, all G1G2), and 7.5 % (6/80, all P[4]P[8]) of pre-vaccination strains. The prevalence of G–P combinations also changed following introduction of the RV vaccine. G1P[8] increased in frequency from 45.5 % (15/33) to 87.5 % (14/16), while G2P[4] decreased from a frequency of 76.5 % (26/34) to the detection in only two strains after Rotarix introduction. We also observed G9P[8] and G9P[4] strains that were not detected in the pre-vaccine period, but which became more prevalent in 2013–2014 and accounted for 57 % (8/14) and 14 % (2/14), respectively (Table 4).

**Table 4:** Prevalence of rotavirus strains before and after rotavirus vaccine introduction

Rotavirus strain	Post vaccination	Pre vaccination	% Proportion difference(95 % CI)	P value
	No(%)	No(%)		
Total strains	48	80		
G1	16(33.3)	33(41.3)	−7.92 % (−25.1; 9.2)	0.366
G2	2(4.17)	34(42.5)	−38.33 % (−50.6; −26.1)	<0.0001
G9	14(29.2)	5 (6.3)	22.9 % (9.0; 36.8)	0.001
G mix^a^	10(20.8)	5 (6.3)	14.6 % (2.0; 27.4)	0.013
P[4]	7(14.6)	27(33.8)	−19.2 % (−33.6; −4.8)	0.009
P[8]	25 (52.1)	21(26.3)	25.8 % (8.7; 42.9)	0.003
P{mix]^b^	11(22.9)	6(7.5)	15.4 % (2.2; 28.6)	0.013
G-P combination				
G1P[8]	14/16(87.5 %)	15/33(45.5 %)	42.0 % (18.6; 65.5)	<0.0001
G2P[4]	2/2 (100)	26/34(76.5 %)	23.5 (9.3; 37.8)	0.001
G9P[8]	8/14(57.1)	0	-	
G9P[4]	2/14 (14.3)	0	-	

G mix^a^, G1G3;G2G9;G3G9, G1G2

P [mix]^b^, P[4]P[8];P[6]P[8];P[8]P[10], P[4]P[8]

## Discussion

Diarrhea remains a major cause of child morbidity and death mostly in low-income countries including Yemen. Rotavirus is the primary cause of severe dehydrating diarrhea in children under 5 years of age. The health and economic burdens are substantial, affecting already overcrowded emergency rooms and hospital inpatient facilities in addition to increasing the financial stresses on poor parents [11, 12].

Following the introduction of RV vaccine, impressive declines in RV and all-cause diarrhea hospitalizations were observed in many countries [13]. This study of the epidemiology of all-cause and rotavirus-specific diarrhea hospitalization is the first to report that rotavirus vaccine introduction in Yemen may have resulted in important epidemiological changes in infant and childhood diarrhea. The study showed an impressive 50 % reduction in all-cause severe dehydrating diarrhea and a statistically significant reduction of rotavirus diarrhea by 48 % in the post-vaccine period (Table 3). After Rotarix vaccine introduction, the prevalence of RVAGE was also significantly reduced in children <12 months of age, who are the most vulnerable (Table 2). Our study also showed that among all children with severe all-cause GE that needed hospitalization, severe RVAGE hospitalization was significantly reduced by 67 % from 43.5 % (796/1828) to 20.1 % (136/675), an OR of 0.33 (95 % CI: 0.27–0.403; *p* < 0.0001) after Rotarix vaccine introduced in Yemen. There was also a significant reduction of 49 % of RVAGE patients visiting the emergency room from 44 % (672/1528) to 28.8 % (67/233), an OR of 0.51 (0.38–0.69; *p* < 0.0001). Despite successful treatment outcome remained unchanged in the pre- and postvaccine periods, we observed a statistically significant reduction with an adjusted OR of 0.615 (95 % CI: 0.400–0.946) of children referred for further intensive care in the post-vaccine period (Table 3). These results suggest reductions in both RV mortality and in the cost of hospitalization may have occurred. Previous reviews of RV vaccine and diarrhea mortality included efficacy studies that showed regional variation, with 91 % prevention of severe RV diarrhea in developed countries, but efficacy as low as 42.2 % in high-mortality countries. The evidence provided by these efficacy studies suggests that RV vaccine has decreased RV-specific and all-cause diarrhea mortality in all regions of the world [14, 15]. In GAVI-eligible countries, including Yemen—one of world’s poorest countries—RV vaccine could prevent an estimated 2.46 million childhood deaths between 2011 and 2030, with an annual decrease of 180,000 at peak vaccine uptake [16].

Rotavirus is most frequently associated with severe dehydrating diarrhea, substantial hospitalization, increased emergency room visits, and increased costs for both hospitals and parents [12]. In this study, in addition to greater than 60 and 49 % reductions of severe RVAGE hospitalization and emergency room visits, respectively, we also observed significant reductions in severe dehydration among severe all-cause and severe RAVGE inpatients. Both showed significant reductions of 58 % from 33.1 % (648/1960) to 17.2 % (117/681), an OR of 0.42 (95 % CI 0.34–0.52), and 55 % from 34.7 % (276/795) to 19.3 % (26/135), an OR of 0.45 (95 % CI: 0.29–0.71), respectively. The introduction of RV vaccine in Yemen opened a new era of vaccine-based intervention for diarrhea control with extensive health and economic benefits. The monitoring of changes in the burden of RV and all-cause diarrhea provides important information to assist health policy-makers in Yemen and neighboring poor countries in the horn of Africa.

Following the introduction of RV vaccine, we observed an important and significant increase in the proportion of children with non-RV AGE at the sentinel hospitals as RVAGE prevalence significantly decreased. We observed an overall increase in the proportion of hospital visits for non-RV AGE to 80.1 % (940/1173) from 59.4 % (2239/3767) in the prevaccine period. Emergency room visits for non-RV AGE increased to 81.3 % (292/359) from 60.8 % (1041/1713), and hospitalization for severe nonRV AGE increased to 79.9 % (539/675) from 56.5 % (1032/1828). A recent study from Nicaragua reported that norovirus (NV) was more commonly detected as a cause of diarrhea while rotavirus became less common following rotavirus vaccine introduction [17]. The study reported that more than 57 % of diarrhea in children under 24 months of age was caused by NV. Another study reported that NV has become the leading viral cause of gastroenteritis in both hospital and community settings in Nicaragua following the implementation of RV vaccination [18]. In Yemen before rotavirus vaccine introduction, NV was detected in 10 % of children from 1 to 60 months of age with AGE [8]. To establish effective management guidelines, epidemiological changes in the causes of childhood diarrhea following introduction of rotavirus vaccine need ongoing surveillance. Our findings of increasing non-RV severe GE could indicate that NV may be increasing and needs close monitoring. Additional preventive interventions that reduce the burden of diarrhea-related morbidity and mortality need consideration. These include promotion of breastfeeding, provision of safe water, and improving sanitation and hygiene in low-income countries, including Yemen, and particularly in communities with poor socioeconomic status.

Worldwide, the most common G genotypes reported are G1, G2, G3, and G9; P[4] and P[8] are the common P genotypes [19, 20]. Before rotavirus vaccine introduction, five G–P combinations: G1P[8], G2P[4], G3P[8], G4P[8], G9P[8] accounted for almost 90 % of rotavirus disease in children worldwide [20]. The only study in Yemen before rotavirus vaccine introduction reported that 55 % of the G–P strains were G1P[8], 21 % were G9P[8], and 12 % were G2P[4], but the study was limited by its restriction to one city (Sana’a) in the highlands. In our study, which was conducted in two cities (Taiz, located at an altitude of 1400 m, and Aden, a coastal city) included surveillance data from a 5-year prevaccine period. We observed that the most prevalent strains were G1P[8], isolated from 15/33 patients (45.5 %), and G2P[4], isolated from 26/34 patients (76.5 %). The G9P[8] strain had not been detected before Rotarix introduction, which may indicate temporal and geographical variations [21].

Many low-income countries in addition to Yemen have added rotavirus vaccine to their national immunization programs. Monitoring vaccine effectiveness against the circulating rotavirus strains and any accompanying changes in strain diversity need continuous active rotavirus surveillance. It is crucial to identify rotavirus strain diversity. Our study revealed a statistically significant 38.3 % (95 % CI: −50.6; −26.1) reduction of G2 (heterotypic) circulating strains in the post-vaccine period even though they are not included in the monovalent G1P[8] vaccine. The G1 strain (partly homotypic) decreased to 33.3 % (16/48) from 41.3 % (33/80), a nonsignificant reduction of 7.9 % (Table 4). Additionally, G9 strains significantly increased in the post-vaccine period, with the emergence of G9P[8] and G9P[4], which were not detected in RV from the pre-vaccine period (Table 4). These findings may indicate that variations in circulating rotavirus strains can occur independently and not as a result of rotavirus vaccine selective pressure. Existing evidence confirms that Rotarix provides good protection against both fully and partly homotypic, and fully heterotypic circulating strains including G2P[4], G9P[4], and G9P[8] [22–24]. A recent systematic review and meta-analysis of published research reports on the distribution of rotavirus strains and strain-specific rotavirus vaccine effectiveness in high- and middle-income Latin America countries showed that rotavirus vaccines (RV1 and RV5) provided adequate protection against RV disease, and that the protection was effective against various homotypic, partly heterotypic, and fully heterotypic rotavirus strains. The review also confirmed that vaccine-induced selective pressure had not occurred. The authors acknowledged the lack of data from low-income countries [25]. A previous study conducted in a low-income setting in Chiapas, the poorest state in Mexico, showed that Rotarix provided adequate protection against severe RVAGE caused by a fully heterotypic strain (G9P[4]), and reported a vaccine effectiveness of 94 % [24]. These findings are reassuring for low-income countries that have recently introduced rotavirus vaccines.

In our study, we observed increase in the prevalence of mixed G and [P] circulating strains after Rotarix vaccine introduction. The G-mix prevalence increased to 20.8 % and [P] mix to 22.9 % (Table 4). Similar observations were reported in a study from Bangladesh [26] that reported a substantial proportion of mixed rotavirus circulating strains of more than 14 %, and exceeding 37 % in 2009–2010 that could have resulted in the generation of unusual combined G[P] circulating strains in 2011–2012. Similar observations have been reported in other countries in the Indian subcontinent [27].

The study has several limitations. It was conducted based on surveillance data collected from two sentinel hospitals, which are not devoid of problems such as missing data. Other limitations are the observational design, and a short post-vaccine period. Lack of information on the vaccination status of children included in the surveillance was a major limitation. The treatment outcomes of 6.8 % of children in the pre-vaccine and 15.8 % in the post-vaccine periods were not known (Table 2), and that may have affected the result of the vaccine impact on the treatment outcomes, particularly diarrhea-related mortality. In addition, follow-up of children with severe AGE who were hospitalized ended on the day of discharge. However, the proportion of children hospitalized with severe all-cause or RVAGE who needed referral for intensive care was significantly reduced (by 38 %) in 2013–2014 (Tables 2 and 3). Finally, the surveillance data were from two governorates, Taiz and Aden, and may not be directly applicable to settings in other governorates of Yemen. However the results provide an important indication of the potential benefits of rotavirus vaccine in other regions of Yemen and the neighboring low-income countries in the horn of Africa because these countries and Yemen have high levels of poverty and high infant and childhood mortality rates.

## Conclusions

This study showed that the introduction of rotavirus vaccine in Yemen significantly reduced both all-cause and RVAGE hospitalization. The proportion of children who were severely dehydrated was also significantly reduced. The prevalence of severe RVAGE was significantly reduced in the post-vaccine period and the seasonal pattern became blunted and less defined with shorter duration. Significant increases in the proportions of children at the sentinel hospitals with non-RV AGE that occurred following RV vaccine introduction may be at least partly due to increasing prevalence of norovirus (NV) AGE.

Scaling-up interventions such as promotion of breastfeeding, improving sanitation facilities, providing a safe water supply, and improved hygiene, including hand washing, are important especially in poor communities to reduce the unexpected rise of non-RV AGE.

Rotavirus circulating strains in the post-vaccine period showed increases in the G and [P] mix strains of more than 20 % each, while G9P[8] and G9P[4] that were not detected in the pre-vaccine period became more prevalent in 2013–2014. Monitoring of rotavirus circulating strains and NV in the post-vaccine era is crucial to set new guidelines to reduce the burden of childhood diarrhea-related mortality and morbidity.

## Abbreviations

RV: Rotavirus
AGE: Acute gastroenteritis
RVAGE: Rotavirus acute gastroenteritis
WHO-EMRO: World Health Organization-Eastern Midditreanian Regional Office
SOP: Standard operating procedures
EIA: Enzyme immune assay
GAVI: Global alliance for vaccine initiative
NV: Norovirus
